# Racial/Ethnic Disparities in Cervical Cancer Screening Services Among Contractors of the Connecticut Breast and Cervical Cancer Early Detection Program

**DOI:** 10.1089/heq.2017.0038

**Published:** 2018-04-01

**Authors:** Morgan A. Pratte, Amy Griffin, Chioma Ogazi, Susan Yurasevecz, Carol A. Blanks, Lisa McCooey, Joy S. Kaufman

**Affiliations:** ^1^ Department of Family Medicine, SUNY Upstate Medical University, Syracuse, New York.; ^2^ Program and Service System Evaluation, The Consultation Center, New Haven, Connecticut.; ^3^ Department of Psychiatry, Yale University, New Haven, Connecticut.; ^4^ Comprehensive Cancer Program, Connecticut Department of Public Health, Hartford, Connecticut.

**Keywords:** cancer screening, health disparities, public health clinics

## Abstract

**Purpose:** Racial/ethnic minority women are at increased risk for cervical cancer. The objective of this study is to use performance management data from the Connecticut Breast and Cervical Cancer Early Detection Program (CBCCEDP) to determine whether race/ethnicity disparities exist in human papillomavirus (HPV) co-testing uptake across CBCCEDP contractors.

**Methods:** Secondary analysis of Connecticut's Minimum Data Elements data for 2013–2015 among 10 contractors participating in the CBCCEDP. Participants included women aged 30–64 years and eligible to receive routine cervical cancer screening services through the CBCCEDP (*n*=5,262). HPV co-testing uptake was compared across contractors and race/ethnicity groups within each contractor using chi-square and Fisher's exact tests as appropriate.

**Results:** Overall, 62.9% of women received HPV co-testing services. Significant differences in co-testing rates were detected between racial/ethnic groups when data were examined across all contractors (*p*<0.001). Black women were least likely to receive co-testing (49.1%), while Hispanic women were most likely to receive co-testing (68.2%). When data were examined at the individual contractor level, significant differences between racial/ethnic groups were observed in 50% of the contractors.

**Conclusions:** This study identified racial/ethnic disparities in uptake of HPV co-testing both overall and within individual contractors involved in the CBCCEDP. These findings will be used to guide program improvement with the goal of increasing quality and consistency of care for all women seeking screening services.

## Introduction

Cervical cancer is one of many health outcomes that disproportionately affects women based on racial/ethnic background. The incidence of cervical cancer is greater in black and Hispanic women compared to white women, even when accounting for socioeconomic status.^1^ Furthermore, racial/ethnic minorities experience greater intervals to both diagnosis and treatment of cervical cancer following screening tests.^2^ The National Breast and Cervical Cancer Early Detection Program (NBCCEDP) is a national public health program that was established by the Centers for Disease Control and Prevention (CDC) upon enactment of The Breast and Cervical Cancer Mortality Prevention Act of 1990, a proposal that aimed to improve cancer screening services to underserved populations.^3^ The purpose of the program is to provide breast and cervical cancer screening and diagnostic services to low-income women who lack adequate health insurance coverage.^4^ State and territorial grantees subcontract with local health clinics and organizations, which in return provide preventive services to priority populations. To be eligible for the program, women must be (1) uninsured or underinsured; (2) at or below 250% of the Federal Poverty Level; and (3) aged 21–64 years for clinical breast examinations and Papanicolaou (Pap) tests or aged 40–64 years for mammograms. Women who have a high insurance deductible of $1,000 or more and meet the age and income eligibility guidelines are also eligible to receive program services.

In addition to providing breast and cervical cancer screening for this medically underserved population, NBCCEDP grantees are responsible for collecting and analyzing data on program performance. Since the program's onset, the CDC established a system among NBCCEDP grantees to monitor client demographics for eligibility and clinical outcomes.^5^ Grantees are required to collect and report on a set of data comprising ∼100 Minimum Data Elements (MDEs) as a record of client services provided.^6^ An NBCCEDP performance management system was implemented in 2006 based on the existing MDE process. DeGroff et al. performed a study to assess the effectiveness of the system, and identified significant improvements in performance measures following its implementation.^5^ NBCCEDP grantees are also required to conduct program evaluation on a regular basis, which allows grantees to identify program needs and areas for improvement. Program evaluation also serves as an opportunity to explore questions about program effectiveness outside the standard activities governed through the performance management system.

The Connecticut Breast and Cervical Cancer Early Detection Program (CBCCEDP) was established in 1995 by the Connecticut Department of Public Health (DPH) through a federal cooperative agreement with the CDC.^7^ The program is available to eligible women at 11 contracted healthcare institutions across Connecticut. Patient eligibility criteria and preventive services provided align directly with those of the overall NBCCEDP. In 2011, the CBCCEDP began a partnership with an external consultant to provide services that address evaluation planning, design, and implementation of targeted projects. One component of the evaluation plan included use of the MDE data to explore service delivery of the CBCCEDP in areas beyond those addressed through the performance management system, allowing the DPH to identify issues specific to the Connecticut-based program.

The CBCCEDP aimed to determine whether differences in cervical cancer screening rates existed between 10 different contracted healthcare institutions and whether there were race/ethnicity disparities in the number and types of services provided. Of particular interest was whether there were disparities in human papillomavirus (HPV) co-testing uptake among women seeking routine cervical cancer screening services. Updated screening guidelines from both the United States Preventive Services Task Force and the American Cancer Society were released in 2012, declaring that women aged 30 through 65 can either be screened every 5 years with Pap testing and HPV testing (subsequently referred to as HPV co-testing) or every 3 years with Pap testing alone.^8,9^ Benefits of using HPV co-testing include higher test sensitivity than Pap testing alone and reduced healthcare costs due to the extension of screening intervals.^10,11^

The purpose of this study is to assess the presence of racial/ethnic differences in HPV co-testing uptake among CBCCEDP contracted healthcare institutions.

## Methods

As previously referenced, the MDEs are a standardized set of ∼100 items required to be collected and reported to the CDC by NBCCEDP grantees.^6^ The MDEs capture patient information such as demographic characteristics, services provided, clinical outcomes, and response times. Connecticut's MDE data set for 2013–2015 was used in this study. The start date of 2013 was selected because this was the first full year in which these cervical cancer screening guidelines were in place (guidelines were initially released in March of 2012). Ten of the 11 current contracted healthcare institutions (subsequently referred to as “contractors”) were included in the analysis; one contractor was excluded because it was added to the CBCCEDP in 2014 and therefore did not have complete data for the period of interest. The patient population included in the analyses consisted of women who met program criteria to receive HPV co-testing from CBCCEDP providers, more specifically, women aged 30–64 years who at the time of their visit were due for routine cervical cancer screening services as indicated by the clinical guidelines previously stated. It is recommended that women with hysterectomies (i.e., those without a cervix) not be routinely screened for cervical cancer, and therefore these women are not considered part of this patient population. Women with missing data on race and ethnicity were excluded from the sample as race/ethnicity was the main covariate of interest in the analysis. This study was determined to be exempt by the Connecticut Department of Public Health Institutional Review Board.

The percentage of women receiving HPV co-testing at the time of their visit was first compared across the 10 contractors (labeled A through J to maintain anonymity). Those who did not receive HPV co-testing had received Pap testing alone. Then co-testing percentages were compared overall between four race/ethnicity groups, which included Non-Hispanic white (NHW), Non-Hispanic black (NHB), Hispanic, and Non-Hispanic Other or Multiracial (NHO). Both race and ethnicity information were self-reported by women. Finally, within each individual contractor, the co-testing percentages were compared between race/ethnicity groups. Chi-square and Fisher's exact tests were conducted as appropriate to determine statistical differences in the utilization of HPV co-testing services between race/ethnicity groups within each contracted healthcare institution. All analyses were performed using SPSS version 23.

## Results

Among the 10 contractors included in the analysis, the initial sample consisted of 14,056 women. Less than half (*n*=6,161) of these women were seeking routine cervical cancer screening services at the time of their visit. Among this subset, 5,365 women were between the ages of 30–64 years. One hundred three women were excluded from the sample due to missing race/ethnicity information, resulting in a final sample of 5,262 women. The mean age of the sample was 46.0 years (SD=9.0 years). The overall race/ethnicity distribution is illustrated in Figure 1, which demonstrates that over half (57.4%) of the sample was Hispanic, about a quarter (24.6%) was NHW, and about 15% was NHB. The remaining 3% was NHO. The number of women in the sample from each contractor ranged from 228 women to 978 women, with a median of 442 women. Contractor-specific characteristics are provided in Table 1. Eight of the 10 contractors primarily serve patients in urban settings, while one contractor serves patients in a rural setting and the other contractor serves patients in a mixture of settings. The mean patient age ranged from 42.8 years at Contractor F to 52.2 years at Contractor C. The race/ethnicity distribution also varied across contractors; more than half (over 50%) of patients were Hispanic at six of the contractors, while Contractor G was only to serve primarily NHB patients (62.2%).

**FIG. 1.**
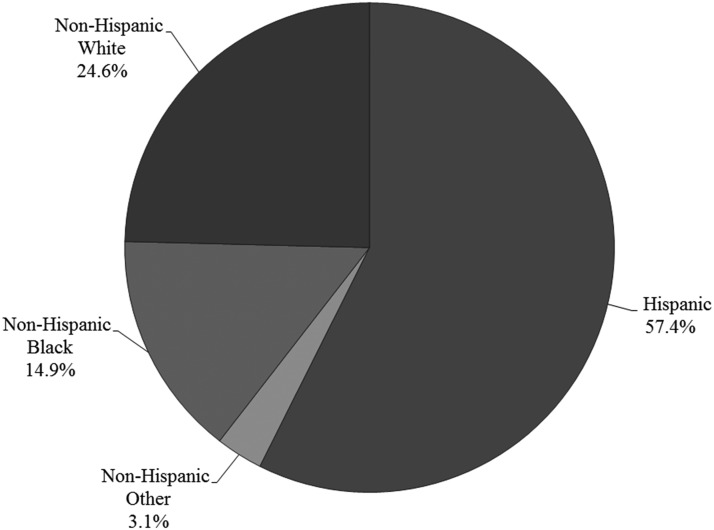
Race/ethnicity distribution of the sample.

**Table 1.** **Contractor Characteristics**

Characteristic	A (*n*=446)	B (*n*=264)	C (*n*=228)	D (*n*=952)	E (*n*=299)	F (*n*=501)	G (*n*=437)	H (*n*=365)	I (*n*=792)	J (*n*=978)
Setting	Urban	Rural	Urban	Mixed	Urban	Urban	Urban	Urban	Urban	Urban
Number of clinics	21	12	6	17	20	2	12	1	1	3
Age, mean (SD)	45.4 (8.9)	48.5 (8.8)	52.2 (7.2)	46.6 (8.5)	48.1 (8.8)	42.8 (8.9)	47.3 (9.3)	47.9 (8.1)	44.9 (8.6)	44.3 (9.2)
Race, *n* (%)										
NHW	112 (25.1)	128 (48.5)	118 (51.8)	185 (19.4)	125 (41.8)	63 (12.6)	42 (9.6)	103 (28.2)	297 (37.5)	122 (12.5)
NHB	23 (5.2)	5 (1.9)	44 (19.3)	153 (16.1)	39 (13.0)	42 (8.4)	272 (62.2)	50 (13.7)	13 (1.6)	141 (14.4)
Hispanic	293 (65.7)	125 (47.3)	51 (22.4)	583 (61.2)	115 (38.5)	383 (76.4)	114 (26.1)	205 (56.2)	476 (60.1)	675 (69.0)
NHO	18 (4.0)	6 (2.3)	15 (6.6)	31 (3.3)	20 (6.7)	13 (2.6)	9 (2.1)	7 (1.9)	6 (0.8)	40 (4.1)

NHB, non-Hispanic black; NHO, non-Hispanic other or multiracial; NHW, non-Hispanic white.

Overall, 62.9% of women received HPV co-testing services at their visit, as opposed to Pap testing alone. Figure 2 presents the co-testing percentages by contractor. Contractor H had the lowest HPV co-testing percentage at 6.6%. Only two additional contractors had percentages below 50% (Contractors B and G). Contractor F had the greatest percentage of women co-tested at 93.2%, followed by Contractor I at 85.6%.

**FIG. 2.**
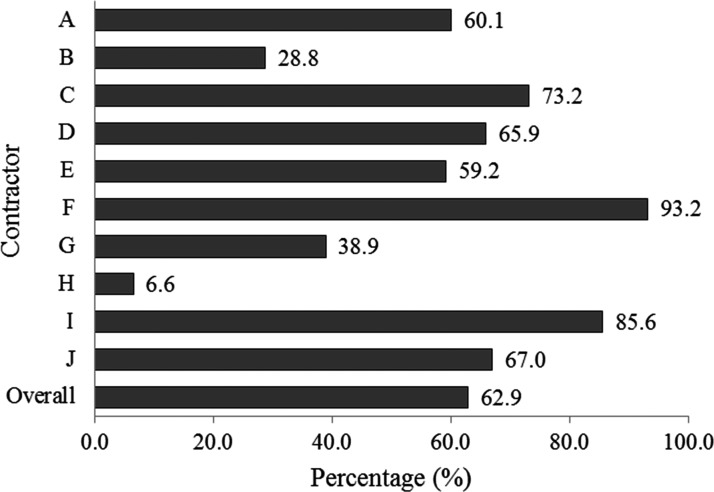
Percentage of women receiving human papillomavirus co-testing by contractor.

Table 2 compares the co-testing percentages by race/ethnicity for the total sample and for each of the contractors individually. Overall, it was determined that co-testing percentages were significantly different between race/ethnicity groups (*p*<0.001). Hispanic women were most likely to receive co-testing (68.2%), followed by NHO (67.9%), NHW (58.2%), and finally NHB women (49.1%). After performing this analysis by an individual contractor, statistically significant differences between race/ethnicity groups were observed for half of these healthcare institutions (A: *p*<0.001; B: *p*=0.005; D: *p*=0.001; E: *p*=0.01; and J: *p*<0.001). Variation was observed among these contractors regarding which race/ethnicity groups were least likely to receive HPV co-testing. However, there were more accounts of lower percentages among NHB and NHW women than Hispanic and NHO women. For example, at Contractor A, 70.0% of Hispanic women received co-testing compared to only 34.8% of NHB and 43.8% of NHW women.

**Table 2.** **Percentage of Women Receiving Human Papillomavirus Co-testing by Race/Ethnicity**

Contractor	Total % (total *N*)	NHW % (total *N*)	NHB % (total *N*)	Hispanic % (total *N*)	NHO % (total *N*)	*p*^a^
A	60.1 (446)	43.8 (112)	34.8 (23)	70.0 (293)	33.3 (18)	<0.001
B	28.8 (264)	36.7 (128)	40.0 (5)	19.2 (125)	50.0 (6)	0.005
C	73.2 (228)	76.3 (118)	68.2 (44)	68.6 (51)	80.0 (15)	0.569
D	65.9 (952)	66.5 (185)	52.9 (153)	68.1 (583)	83.9 (31)	0.001
E	59.2 (299)	48.8 (125)	71.8 (39)	63.5 (115)	75.0 (20)	0.011
F	93.2 (501)	95.2 (63)	90.5 (42)	93.2 (383)	92.3 (13)	0.725
G	38.9 (437)	26.2 (42)	39.0 (272)	43.9 (114)	33.3 (9)	0.240
H	6.6 (365)	5.8 (103)	8.0 (50)	6.8 (205)	0.0 (7)	0.897
I	85.6 (792)	83.5 (297)	84.6 (13)	86.8 (476)	100.0 (6)	0.548
J	67.0 (978)	48.4 (122)	53.9 (141)	72.7 (675)	72.5 (40)	<0.001
Overall	62.9 (5262)	58.2 (1295)	49.1 (782)	68.2 (3020)	67.9 (165)	<0.001

^a^ *p*-Value for χ^2^ or Fisher's exact test.

## Discussion

These findings reveal noteworthy discrepancies in cervical cancer screening practices across the CBCCEDP partnership of contracted healthcare institutions. Uptake of HPV co-testing services among individual contractors ranged considerably from 6.6% to 93.2%. Furthermore, half of the contractors under assessment demonstrated significant differences in HPV co-testing percentages by race/ethnicity groups. Overall, NHB women were least likely to receive HPV co-testing services, almost a 20% difference when compared to Hispanic women, who were most likely to receive HPV co-testing.

Although the American Cancer Society (ACS) guidelines declare HPV co-testing every 5 years as the preferred screening method among women aged 30–65 years,^12^ many providers and patients are reluctant to use HPV co-testing and/or to extend screening intervals. In a survey among primary care providers who serve low-income, underserved populations, the majority of respondents indicated that co-testing is beneficial, but less than half routinely used co-testing in practice.^13^ In addition, providers report that their primary concerns with regard to lengthening screening intervals are that patients may miss annual visits for other screening tests, patients losing contact with the healthcare system, and risk of liability if the patient's subsequent results are abnormal.^13^ Likewise, patients are hesitant to adopt current guidelines. Research indicates that patients report that a higher level of perceived risk, having an abnormal screening history, and reduced frequency of care reduce their willingness to accept longer screening intervals.^14^ In addition, lack of patient knowledge of screening guidelines and practices has been found to be a major barrier to adherence to current recommendations.^15^ Educational interventions aimed at women's healthcare providers have been shown to be promising in increasing both the adoption of extended screening intervals and utilization of HPV co-testing.^16,17^ Given that provider recommendation is often a leading factor in a patient's decision to follow through with screening tests, implementing provider awareness programs to promote co-testing could be a first step in increasing uptake.

Although patient and provider barriers are likely to contribute to the inconsistencies in HPV co-testing utilization across contracted healthcare institutions identified in this analysis, there may also be larger-scale challenges at play as well. Subramanian et al. conducted a study to identify factors associated with variation in screening practices across NBCCEDP grantees and found that the size of the eligible patient population, cost of service delivery, and urban versus rural mix of patients all impacted practice.^18^ Upon identifying extensive variation in HPV co-testing uptake among the Connecticut contractors, it may be important to explore the organizational and systems-level factors that could be contributing to these contractor-level differences. While Connecticut is a relatively small state, each contractor does have unique characteristics, including population served and available resources in the local community. Finally, systemic issues need to be considered in understanding screening rates, for example, clinics may be tied to practice policies enforced by a governing body that dictates the types and intervals in which screening services are available to their patients.

While the factors listed above may contribute to the differences in screening practices between individual healthcare institutions, there is a lack of research on whether they are associated with the racial/ethnic disparities detected within individual CBCCEDP contractors. In a public health program aimed at providing services to minority and underserved populations, it is unclear why discrepancies in care across different race/ethnicity groups exist. One possible explanation could be the way in which providers approach shared decision-making based on sociodemographic characteristics. When discussing the need for cancer screening, Bao et al. identified that patients were treated differently by the same physicians, what they considered to be “within-physician” differences, depending on the patient's race or education level.^19^ Additional studies have indicated that black patients experience less participatory encounters with their providers when compared to white patients.^20,21^ Given these disparities, there is promising evidence that interventions aimed at improving shared decision-making (e.g., enhancing communication skills and utilizing electronic decision aids) among disadvantaged populations could help to address health inequalities.^22^ One of the overarching goals of Healthy People 2020 is to “achieve health equity, eliminate disparities, and improve the health of all groups.”^23^ To make progress toward this essential goal within the CBCCEDP, it is critical to follow up on the findings of this study by investigating and addressing the factors underlying this apparent inequity.

Certain limitations should be acknowledged when interpreting the findings of this study. Detailed information on contractor, provider, and patient characteristics was not available from the existing MDE data set. Therefore, we were unable to assess the influence of potential confounding factors such as patient volume, costs of service delivery, and urban/rural patient mix at the contractor level, within- or between-provider differences, or insurance status, education, and other socioeconomic indicators at the patient level. Future studies should seek to gather these data as these variables would not only supplement what is currently accessible through the MDE data system but would also provide rich information that can be used to enhance program performance.

The initial results from this evaluation were presented to staff from the Connecticut contractors at a cross-site meeting. Follow-up site visits were conducted in which potential contributors to some of the cross-site differences were detected, including variations in methods for scheduling women for screening tests and the availability of screening clinic days. Using the outcomes of this evaluation, CBCCEDP staff members have had discussions with contractors regarding strategies to integrate the screening program into their workflow with the goal of improving the work processes and increasing screening rates. One strategy recently undertaken by the CBCCEDP was the implementation of a team-based approach to patient-centered care, which establishes a comprehensive navigation team for each contracted healthcare institution. The navigation team seeks to connect underserved women in the community with the healthcare services that they need. The CBCCEDP staff continue to work with contractor teams to consider program improvement strategies such as documenting workflow and policies, sharing best practices, and building upon existing evaluation efforts.

This study identifies areas for possible expansion of this work. Given that research has shown that patient and provider beliefs and knowledge impact co-screening rates,^13–16^ understanding these issues locally will inform the types of educational interventions that are needed to impact a system. As previously mentioned, additional practice-level data could be collected regarding patient population characteristics (e.g., demographics, volume, and rural/urban mix), provider characteristics, costs of service delivery, and cancer screening policies to expand the opportunities for understanding the issues that may impact rates of co-testing. Furthermore, it is essential to enhance our understanding of the potential reasons for the disparities revealed in this study, with the goal of eradicating these differences and providing equal access for all eligible women. This enhanced information is vital in informing the development of patient and/or provider education, outreach activities, and practice policy changes aimed at increasing rates of co-testing for all women.

## Conclusion

Performance management data were used to evaluate cervical cancer screening practices, specifically the uptake of HPV co-testing, among contractors of the Connecticut Breast and Cervical Cancer Screening Program. Findings from the analysis show that HPV co-testing usage varies greatly between sites. Furthermore, this is the first study to our knowledge that assessed HPV co-testing uptake by race/ethnicity. Significant differences between race/ethnicity groups were revealed, in which NHB women were least likely to be screened by HPV co-testing and Hispanic women were most likely to undergo this screening method. These findings will be used to guide program improvement through continued evaluation of the factors underlying these disparities and development of interventions aimed at ensuring that all populations receive equal and quality care.

## Abbreviations Used

CBCCEDP: Connecticut Breast and Cervical Cancer Early Detection Program
HPV: human papillomavirus
MDE: minimum data element
NBCCEDP: National Breast and Cervical Cancer Early Detection Program
NHB: non-Hispanic black
NHO: non-Hispanic other or multiracial
NHW: non-Hispanic white
